# Three-dimensional quantitative evaluation of hypertension-induced aortic fibre remodelling based on multiphoton microscopy: a cross-age perspective

**DOI:** 10.1098/rsos.251339

**Published:** 2025-09-03

**Authors:** Nannan Wang, Rongli Zhang, Anni Zhao, Jia Yu, Yufeng Gao, Xuheng Sun, Wanwen Chen, Na Xiao, Feng Xiang, Wei Zheng, Zhanyi Lin, Hui Li

**Affiliations:** ^1^ Research Center for Biomedical Optics and Molecular Imaging, Shenzhen Key Laboratory for Molecular Imaging, Guangdong Provincial Key Laboratory of Biomedical Optical Imaging Technology, Shenzhen Institute of Advanced Technology, Chinese Academy of Sciences, Shenzhen 518055, People's Republic of China; ^2^ Key Laboratory of Biomedical Imaging Science and System, Chinese Academy of Sciences, State Key Laboratory of Biomedical Imaging Science and System, Shenzhen 518055, People's Republic of China; ^3^ Department of Diagnostic Radiology, Li Ka Shing Faculty of Medicine, The University of Hong Kong, Pok Fu Lam, Hong Kong; ^4^ Department of Cardiology, Guangdong Cardiovascular Institute, Guangdong Provincial People's Hospital, Guangdong Academy of Medical Sciences, Guangzhou 510080, People's Republic of China; ^5^ Jinfeng Laboratory, Chongqing 401329, People's Republic of China; ^6^ Jihua Laboratory, Foshan 528000, People's Republic of China

**Keywords:** hypertension, multiphoton microscopy, aortic fibre remodelling, 3D grey-level co-occurrence matrix, cross-age evaluation

## Abstract

Hypertension is the primary cause of cardiovascular diseases, and its worldwide prevalence has continued to increase recently. Aortic fibre remodelling is critical in the development of hypertension and is strikingly age-related. However, the underlying microlevel variations remain unknown. This study quantitatively evaluated the hypertension-induced microstructural remodelling of aortic fibres from a cross-age perspective by combining label-free multiphoton microscopy (MPM) imaging with a three-dimensional (3D) grey-level co-occurrence matrix (GLCM) algorithm. First, MPM imaging of aortic collagen and elastin fibres was performed on spontaneously hypertensive rats and Wistar–Kyoto controls across three critical age stages (prehypertension, developing hypertension and stable hypertension) and two aortic segments (abdominal aorta and thoracic aorta). Subsequently, the 3D GLCM texture features that were significantly correlated with hypertension or age-related hypertension were identified. By deciphering these features, we revealed quantitative details of hypertension-induced aortic remodelling, hypertension-accelerated aortic ageing and the heterogeneous response of different aortic segments to hypertension from the perspective of the fibre microstructure. The proposed method and derived findings may shed new light on the mechanism of age-related hypertension and contribute significantly to the research on cardiovascular diseases.

## 1. Introduction

Hypertension is a major cause of morbidity and mortality in the older population and a significant risk factor for cardiovascular diseases [1]. Affected by unhealthy habits, such as smoking, drinking and irregular diet, as well as numerous environmental factors, the worldwide prevalence of hypertension and its complications is continually increasing [2]. Aortic stiffening, which is closely related to variations in the three-dimensional (3D) anisotropic structures of collagen and elastin fibres within the aortic walls [3], has been recently recognized as both a cause and consequence of hypertension, especially in middle-aged and older individuals [4]. This highlights the importance of fibre remodelling in the development of age-related hypertension. Investigating the hypertension-induced 3D remodelling of aortic fibre microstructures from a cross-age perspective may provide novel insights into the prevention and control of hypertension and enhance our understanding of the occurrence and development mechanisms of cardiovascular diseases.

Cardiovascular magnetic resonance (CMR) imaging has been extensively used to detect early stage structural changes in the aortic wall [5]. Other medical imaging modalities, such as computed tomography (CT), invasive coronary angiography (ICA), intravascular ultrasound (IVUS) and optical coherence tomography (OCT) [6–8], as well as emerging and promising photoacoustic imaging techniques, such as photoacoustic tomography [9] and intravascular photoacoustic imaging [10], have been widely used in addition to CMR to characterize morphological and structural variations in the aorta. Notwithstanding the value of these technologies and methods, the limited spatial resolution and/or non-specificity to fibre components substantially restricts their application in evaluating the 3D remodelling of aortic fibre microstructures. Traditional histological inspection based on tissue sections with specific staining, such as Verhoeff–Van Gieson (VVG) staining for labelling elastin fibres and Masson’s trichrome staining for highlighting collagen fibres, remains the mainstream method for revealing aortic fibre microstructures. Using this technique, elastin fibre fragmentation [11], increased collagen content and tunica media thickening [12,13] in the aortic walls were observed during hypertension. However, histological inspection is limited to two-dimensional (2D) characterization, and sample preparation is complicated, time-consuming (3–5 days), and readily causes fibre deformation [14].

In recent decades, multiphoton microscopy (MPM) based on second-harmonic generation (SHG) and two-photon excitation fluorescence (TPEF) has emerged as a powerful tool for aortic fibre evaluation. This technology directly delineates the collagen and elastin fibres in 3D using endogenous SHG and TPEF signals, respectively. Empowered by subcellular resolution, intrinsic optical sectioning capacity (the nonlinear excitation process in MPM inherently rejects the out-of-focus background by confining signal generation to the focal volume) and label-free imaging ability, fibre microstructures comparable to details unveiled by standard histological inspection can be obtained without any special sample preparation. Moreover, MPM has a superior penetration depth and reduced photobleaching and photodamage [15,16]. Owing to these distinct advantages, this technology has been widely used to evaluate fibre remodelling in studies on arterial ageing [17–19] and various cardiovascular diseases, such as arterial aneurysm [20,21], Marfan syndrome [22,23] and atherosclerosis [24]. Furthermore, algorithms such as fractal dimension, directional analysis [18], fast Fourier transform [25,26] and grey-level co-occurrence matrix (GLCM) [24,27] have been used in these studies to quantitatively characterize fibre textures for more objective and automatic assessment. These studies provide novel insights into the mechanisms underlying vascular remodelling and disease progression. Despite the recent boom in MPM for deciphering vascular fibres, it is noteworthy that hypertension-induced variation in fibre microstructures remains a virtually untapped area, let alone from a cross-age perspective.

In this study, we propose a quantitative evaluation of aortic fibre remodelling induced by age-related hypertension by combining MPM imaging with the classical spatial texture characterization algorithm 3D GLCM. The 3D microstructures of elastin and collagen fibres within the aortas harvested from spontaneously hypertensive rats (SHR) were investigated. Two aortic segments—the abdominal aorta (AA) and thoracic aorta (TA)—were analysed across three critical stages: prehypertension, developing hypertension and stable hypertension. By comparing the SHR rats with age-matched Wistar–Kyoto (WKY) normotensive controls, we identified a series of fibre texture features that were significantly correlated with hypertension or age-related hypertension. Based on these features and the derived information, we revealed the quantitative details of hypertension-induced aortic fibre remodelling from a cross-age perspective and provided new evidence demonstrating the accelerating effect of hypertension on ageing and heterogeneity across different aortic segments. The proposed method, as well as the derived indicators and information, may offer new insights into the mechanism of age-related hypertension and would be substantially beneficial for research on various cardiovascular diseases.

## 2. Material and methods

### 2.1. Animal model

SHR, which are typical hypertension model rats, were used in this study and analysed at three critical stages: prehypertension (3 weeks old), developing hypertension (12 weeks old) and stable hypertension (44 weeks old) [28,29]. Age-matched WKY rats served as normotensive controls. For each experimental group (defined by strain and age), 3−4 rats were included (comprising 4 SHR rats across all age stages, 4 WKY rats for the 3- and 12-week-old groups and 3 WKY rats for the 44-week-old group; electronic supplementary material, table S1). The rats were purchased from Beijing Vital River Laboratory Animal Technology Co. (Beijing, China). All rats were male and genetically screened to establish a stable model with low phenotypic variability, eliminating sex-related biases and minimizing sample size-dependent variation.

### 2.2. Blood pressure measurement

To evaluate the hypertension model, the blood pressure of SHR and WKY rats was measured while awake by tail-cuff plethysmography using a non-invasive caudal artery systolic blood pressure (SBP) measurement and analysis system (ZS-Z, Zhongshi Dichuang Technology Development Co.). For accurate SBP measurement, 30 min acclimation followed by 30 min heating (34℃) were allowed to ensure the rats were in a quiet and relaxed state. Three consecutive stable measurements were averaged to obtain the final SBP for each rat. For 3-week-old rats, SBP values were not measured because their tails were too thin to wear the tail cuff.

### 2.3. Aortic tissue harvest

**Figure 1.**
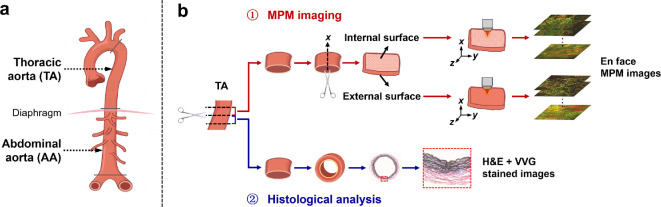
(a) Anatomical sampling sites of the thoracic aorta (TA) and abdominal aorta (AA). (b) Subsequent specimen preparation protocols (exemplified by TA). MPM, multiphoton microscopy; H&E, haematoxylin and eosin; VVG, Verhoeff–Van Gieson.

The AA and TA, which are two aortic segments crucial for blood circulation, were analysed. They were harvested from SHR and WKY rats sacrificed by CO_2_ euthanasia, and perivascular adipose tissues were removed from the outer walls. For each aortic segment, the harvested tissue was transversely sectioned into two adjacent circumferential sections. The paired specimens were then allocated as follows: one portion was fixed for histological analysis and its anatomically matched counterpart was immediately prepared for multiphoton imaging. The anatomical sampling sites and subsequent specimen preparation protocols are illustrated in figure 1. Consistent anatomical locations were maintained during tissue collection to ensure robust histological comparison across all specimens.

### 2.4. Histological analysis

To assess the animal model and evaluate the overall changes in the aortic wall induced by hypertension and ageing, paraffin sections of the aortic segments were prepared and imaged in cross-sectional view. Haematoxylin and eosin (H&E) staining and VVG staining were used to reveal the overall histology of the aortic wall and organization of elastin fibres, respectively. Because tunica media thickening is recognized as a characteristic of hypertension [30], the media thickness of each aortic segment was measured to evaluate the hypertension model used in our study. The tunica media features layered elastin fibres (i.e. elastic laminae [ELs]) to support smooth muscle cells [31] and thus can be easily identified in the VVG-stained sections. Therefore, we directly measured the media thickness in the section images using the CaseViewer software (3DHISTECH). For each aortic segment, 4−5 randomly selected regions were measured and averaged to obtain the final media thickness (mean ± s.d.). Related technical services were provided by Servicebio Technology Co. (Wuhan, China).

### 2.5. MPM imaging

For the MPM imaging of the aforementioned aortic segments, a commercial system (A1R-MP, Nikon) equipped with a Ti: sapphire laser (MaiTai eHP DeepSee, Spectra-Physics) was used. By tuning the laser wavelength to 790 nm, the SHG signals of the collagen fibres and the TPEF signals of the elastin fibres within the aortic wall were simultaneously excited. A 25×/NA 1.1 water immersion objective (N25X-APO-MP, Nikon) was used to focus the laser beam onto the tissue and collect the signals. Afterwards, the SHG (382.5−407.5 nm; ET395/25 m-2p, Chroma) and TPEF (425–495 nm; T425lpxr and T495lpxr, Chroma) signals were separately recorded in different detection channels. The images were acquired in 3D with a voxel resolution of 0.5 μm × 0.5 μm × 3 μm (i.e. the axial [*x*], circumferential [*y*] and radial [*z*] step distances are 0.5 μm, 0.5 μm and 3 μm, respectively). The field of view is 500 μm× 500 μm. Real-time laser power compensation was applied to counteract the depth-dependent signal attenuation, with the maximum power maintained below 40 mW.

For each aortic segment, en face imaging was performed on both internal and external surfaces. To facilitate two-sided imaging, the harvested aortic segments were fully opened, flattened and sandwiched between two glass coverslips, and the tissue was soaked in phosphate-buffered saline (pH 7.4) during imaging. The intima and media were imaged from the internal side, whereas the adventitia was imaged from the external side. To ensure clear layer-specific characterization, the imaging depths were carefully controlled to avoid the transitional zone between the media and adventitia. As the thickness of the aortic wall significantly varies across age, the imaging depth was set to 60 μm for TA and AA of 12- and 44-week-old rats in both sides of imaging, whereas this was shortened to 30 μm for 3-week-old rats. For age-matched WKY and SHR rats, identical depth ranges were maintained, as thickness variations between strains were minimal relative to age-related differences. Although the imaged depths covered approximately 30−90% of the intima–media or adventitia thickness (varying owing to individual differences and between aortic segments/strains), these partial-thickness images adequately represent the histological architecture owing to the structural homogeneity within each layer. This strategy ensures a robust comparison in subsequent quantitative analyses. At least three randomly selected sites were imaged on each side. The specific sample sizes for each group are summarized in electronic supplementary material, table S1.

### 2.6. 3D GLCM analysis

**Figure 2.**
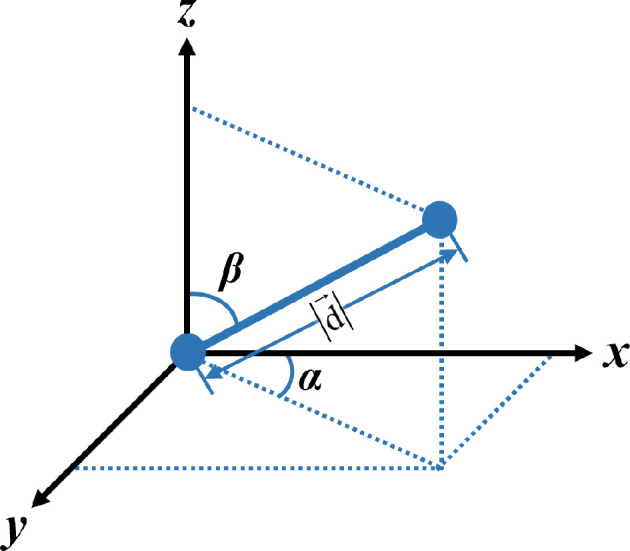
Definitions of key parameters determining the relative positions of a pair of voxels (blue dots) in the three-dimensional (3D) grey-level co-occurrence matrix (GLCM) analysis.

The classical spatial texture characterization algorithm, 3D GLCM, was used to quantify the aortic fibre remodelling induced by age-related hypertension. The details of the algorithm are provided in previous reports [32–34]. In brief, for a given 3D image with *G* grey levels, the 3D GLCM is a matrix that records the second-order joint probability $p(i,j)|\vec{d},G$ of any two voxels with grey levels of *i* and *j* (0 ≤ *i*, *j* ≤ *G*−1), respectively, and apart from each other with a displacement of $\vec{d}$. As shown in figure 2, $\vec{d}$ is a vector defined by distance,$\left|\vec{d}\right|$, and direction, (*α*, *β*), where *α* is a azimuth angle and *β* is a zenith angle.

Based on the 3D GLCM, ten texture features (table 1) [32–34] were calculated. To minimize the effect of intensity variations among images on texture feature values, the contrast of the obtained MPM images was enhanced using the linear image intensity adjustment (implemented via MATLAB [MathWorks] function ‘imadjust’) before 3D GLCM analysis. This method was selected because it balances detail enhancement and noise suppression more effectively than nonlinear alternatives, such as histogram equalization and image sharpening. The image enhancement ensures that the texture feature value reflects only the microstructural characteristics of the collagen and elastin fibres. Also by MATLAB programming, the 3D GLCM as well as the ten texture features were calculated with four distances, $\left|\vec{d}\right|$ = 3, 4, 5, and 6 voxels, and 13 directions, (*α*, *β*) = (–, 0°), (0°, 45°), (45°, 45°), (90°, 45°), (135°, 45°), (0°, 90°), (45°, 90°), (90°, 90°), (135°, 90°), (0°, 135°), (45°, 135°), (90°, 135°) and (135°, 135°). Here, ‘–’ denotes arbitrary angle because when *β* goes to 0°, *α* will be unavailable (figure 2). For subsequent statistical analysis, the values of each texture feature calculated with different distances and directions were averaged to enhance the analytical robustness and data representativeness.

**Table 1.** Three-dimensional (3D) grey-level co-occurrence matrix (GLCM) texture features [32–34].

texture feature	formula	description
entropy	$-\sum_{i=0}^{G-1}\sum_{j=0}^{G-1}p\left(i,j\right)log\left(p\left(i,j\right)\right)$	characterizes the randomness/variability in neighbourhood intensity values
max probability	Max(p(i,j))	quantifies the occurrence probability of the most predominant pixel pair, or the textural reproducibility
sum mean	$\frac{\sum_{i=0}^{G-1}\sum_{j=0}^{G-1}\left(i+j\right)p\left(i,j\right)}{2}$	reflects the average intensity of pixel pairs, highlighting the overall brightness level.
energy	$\sum_{i=0}^{G-1}\sum_{j=0}^{G-1}p{\left(i,j\right)}^{2}$	measures textural uniformity and thickness
contrast	$\sum_{k=0}^{G-1}k^{2}\left\{\sum_{i=0}^{G-1}\sum_{j=0}^{G-1}p\left(i,j\right)\right\}$	quantifies textural sharpness
cluster prominence	$\sum_{i=0}^{G-1}\sum_{j=0}^{G-1}{\left(i+j-\mu_{i}-\mu_{j}\right)}^{4}p\left(i,j\right)$	characterizes the skewness and asymmetry of GLCM
cluster shade	$\sum_{i=0}^{G-1}\sum_{j=0}^{G-1}{\left(i+j-\mu_{i}-\mu_{j}\right)}^{3}p\left(i,j\right)$	quantifies the skewness and uniformity of GLCM, reflecting the local intensity variation
correlation	$\frac{\sum_{i=0}^{G-1}\sum_{j=0}^{G-1}\left(ij\right)\cdot p\left(i,j\right)-\mu_{\text{i}}\mu_{j}}{\sigma_{i}\sigma_{j}}$	characterizes the linear dependency of grey levels on those of neighbouring pixels, reflecting the local correlation of grey levels
variance	$\begin{array}{r} \sum_{i=0}^{G-1}\sum_{j=0}^{G-1}{\left(i-\mu_{i}\right)}^{2}\cdot p\left(i,j\right)+\sum_{i=0}^{G-1}\sum_{j=0}^{G-1}{\left(j-\mu_{j}\right)}^{2}\cdot p\left(i,j\right) \end{array}$	measures the dispersion (with regard to the mean) of the grey level distribution or the heterogeneity of the image
homogeneity	$\sum_{i=0}^{G-1}\sum_{j=0}^{G-1}\frac{1}{1+{\left(i-j\right)}^{2}}p\left(i,j\right)$	measures the smoothness of the grey level distribution

Notes: $\mu_{i}=\sum_{i=0}^{G-1}\sum_{j=0}^{G-1}i\cdot p\left(i,j\right),\;\mu_{j}=\sum_{i=0}^{G-1}\sum_{j=0}^{G-1}j\cdot p\left(i,j\right),\;\sigma_{i}=\sqrt{\sum_{i=0}^{G-1}\sum_{j=0}^{G-1}{\left(i-\mu_{i}\right)}^{2}p\left(i,j\right)},\;\;and\;\sigma_{j}=\sqrt{\sum_{i=0}^{G-1}\sum_{j=0}^{G-1}{\left(j-\mu_{j}\right)}^{2}p\left(i,j\right)}.$

### 2.7. Statistical analysis

All statistical analyses were performed using the GraphPad Prism software. The normality of distribution for all datasets was assessed using the Shapiro–Wilk test. For comparisons between SHR and WKY rats of the same age, the Student’s *t*‐test was applied to normally distributed data, whereas the Mann–Whitney *U*-test was used for non-normally distributed data. For comparisons across age groups (3, 12 and 44 weeks old) within the same rat strain, one-way analysis of variance (ANOVA) with Tukey’s post-hoc test and the Kruskal–Wallis test with Dunn’s post-hoc test were used for normally and non-normally distributed data, respectively. Statistical significance was defined as *p* < 0.05.

## 3. Results

### 3.1. Hypertension model assessment based on SBP measurement and routine histological analysis

**Figure 3.**
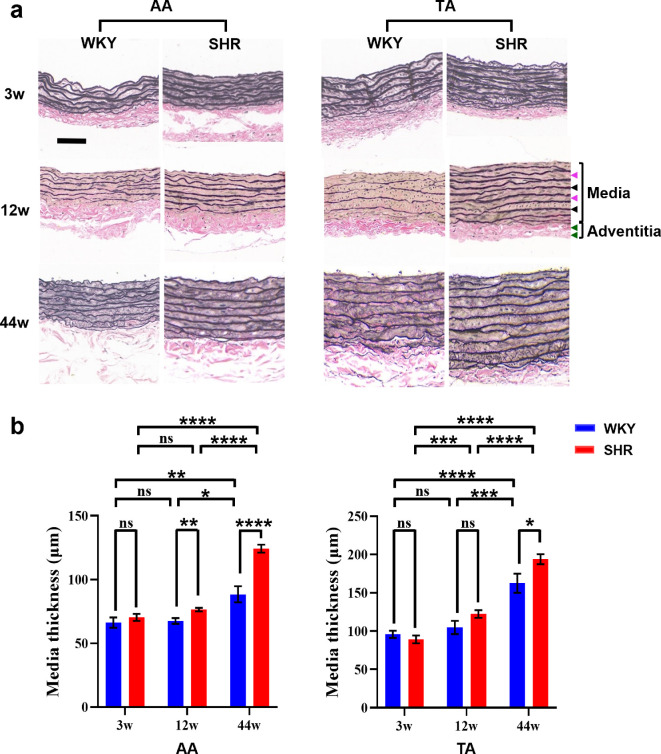
Histological analysis of aortic walls from the spontaneously hypertensive rats (SHR) and the Wistar–Kyoto (WKY) controls of different ages. (a) Representative histological images (combined staining with haematoxylin and eosin [H&E] and Verhoeff–Van Gieson [VVG]; cross-sectional view). Magenta and black arrowheads indicate smooth muscle-rich layers (SMLs) and elastic laminae (ELs) in the tunica media, respectively. Green arrowheads indicate collagen fibres in the tunica adventitia. Scale bar: 60 μm. (b) Media thickness. Data are presented as the mean ± standard error of the mean. ns: no significant difference; *p < 0.05; **p < 0.01; ***p < 0.001; ****p < 0.0001. AA, abdominal aorta; TA, thoracic aorta; 3w, 3 weeks old; 12w, 12 weeks old; 44w, 44 weeks old.

To investigate hypertension-induced aortic fibre remodelling, SHR were compared with WKY controls. We first confirmed the hypertensive phenotype in SHR through SBP measurement, with significantly elevated pressures measured at both 12 weeks (146 ± 8.29 mm Hg in SHR versus 97.2 ± 5.76 mm Hg in WKY) and 44 weeks (214.7 ± 24 mm Hg in SHR versus 162 ± 16.7 mm Hg in WKY). In addition, routine histological analysis revealed significantly thicker tunica media in SHR relative to that in WKY controls, particularly at the stable hypertension stage (44 weeks) (figure 3). This structural alteration, which is consistent with observations in hypertensive patients [35,36], further validated our experimental model. Moreover, thickening of the tunica media with ageing was also observed, which was even more pronounced than hypertension-induced thickening (figure 3), suggesting a potential synergistic relationship between hypertension and ageing.

### 3.2. Aortic fibre microstructures in normal and hypertensive rats of different ages visualized by MPM imaging

**Figure 4.**
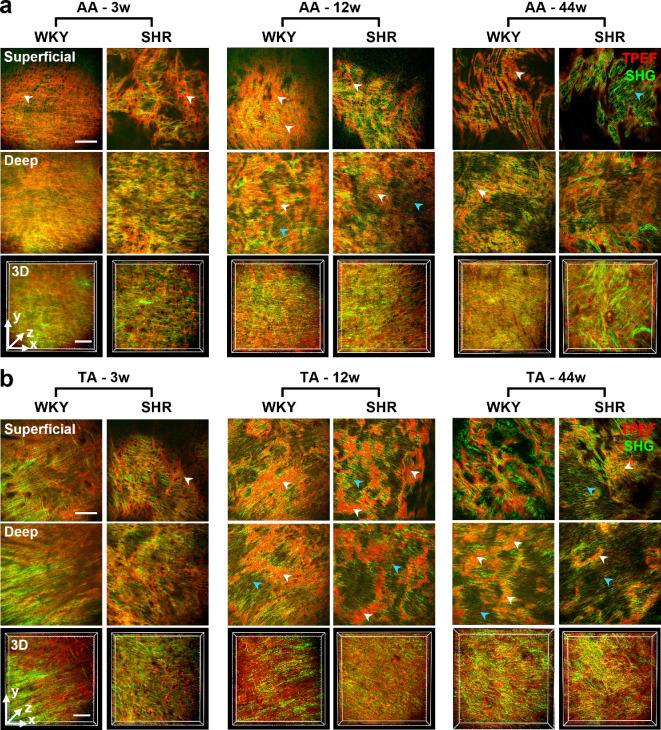
En face multiphoton microscopy (MPM) imaging of the internal surface of the aortic wall revealed the microstructures of intima–media fibres of the (a) abdominal aorta (AA) and (b) thoracic aorta (TA) in spontaneously hypertensive rats (SHR) and Wistar–Kyoto (WKY) controls of different ages: 3w, 3 weeks old; 12w, 12 weeks old; 44w, 44 weeks old. Representative two-dimensional (2D) single-slice images from the superficial layer (depth: 0−10 μm for 3w rats, 0−20 μm for 12w and 44w rats) and the deep layer (depth: 10−30 μm for 3w rats, 20−60 μm for 12w and 44w rats), as well as the three-dimensional (3D) reconstruction across the two layers (depth range: 0−30 µm for 3w rats, 0−60 µm for 12w and 44w rats) were presented. Collagen and elastin fibres were visualized via second-harmonic generation (SHG, green) and two-photon excitation fluorescence (TPEF, red) signals, respectively. White and cyan arrowheads indicate elastic laminae (ELs) and smooth muscle-rich layers (SMLs), respectively. Scale bars: 100 μm (applied to all images).

**Figure 5.**
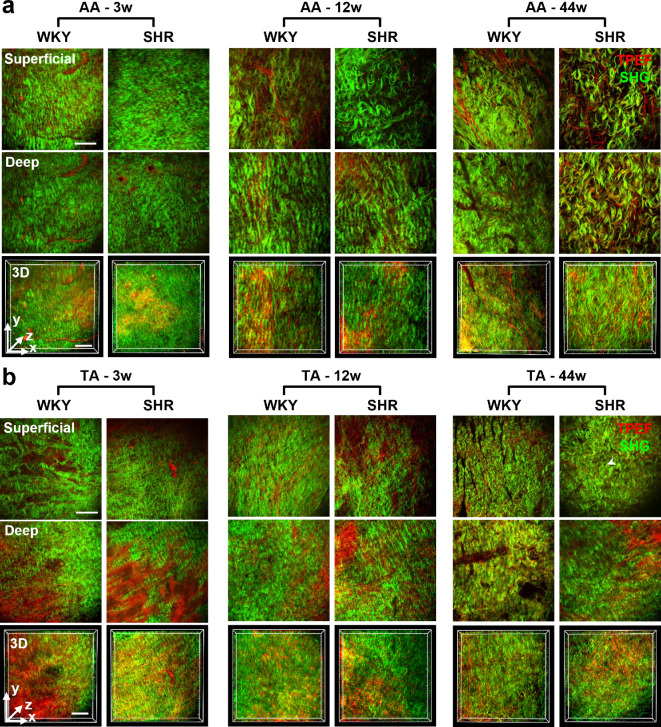
En face multiphoton microscopy (MPM) imaging of the external surface of the aortic wall revealed the microstructures of adventitia fibres of the (a) abdominal aorta (AA) and (b) thoracic aorta (TA) in spontaneously hypertensive rats (SHR) and Wistar–Kyoto (WKY) controls of different ages: 3w, 3 weeks old; 12w, 12 weeks old; 44w, 44 weeks old. Representative two-dimensional (2D) single-slice images from the superficial layer (depth: 0−10 μm for 3w rats, 0−20 μm for 12w and 44w rats) and the deep layer (depth: 10−30 μm for 3w rats, 20−60 μm for 12w and 44w rats), as well as the three-dimensional (3D) reconstruction across the two layers (depth range: 0−30 µm for 3w rats, 0−60 µm for 12w and 44w rats) were presented. Collagen and elastin fibres were visualized via second-harmonic generation (SHG, green) and two-photon excitation fluorescence (TPEF, red) signals, respectively. Scale bars: 100 μm (applied to all images).

After demonstrating the hypertension model and general variation of the aorta induced by age-related hypertension, the corresponding microstructural variations were assessed using en face MPM imaging of both the internal and external surfaces of the aortic wall (figures 4 and 5). From the internal side, the intima and media were imaged, whereas from the external side, the adventitia structures were captured. Collagen and elastin fibres were visualized via SHG and TPEF signals, respectively. In general, the content of elastin fibres was relatively higher than that of the collagen fibres in the internal wall of the aorta, whereas the external wall was predominantly composed of collagen fibres, with a few elastin fibres.

Based on the above overall cognition, we investigated the microstructural details. Specifically, consistent with histological findings (figure 3), the tunica intima remained unidentifiable in the MPM images (figure 4). However, the two main structures of the tunica media, smooth muscle-rich layers (SMLs) and ELs, can be easily identified based on the relative content of collagen and elastin fibres. SMLs featured sparse and regular elastin fibres with comparable collagen fibres (cyan arrowheads in figure 4), whereas ELs were composed of abundant and dense elastin fibres (white arrowheads in figure 4). Comparing the MPM images of WKY and SHR rats (figure 4), the most evident difference was observed in ELs at the prehypertension stage (3 weeks) in both the AA and TA. ELs in the WKY group presented a plate-like structure with uniformly oriented fibres, whereas the ELs in the SHR group exhibited a reticular structure formed by disoriented fibres. At the hypertension development stage (12 weeks), a similar difference was observed in AA in the superficial layers. For the deep layers, the ELs of WKY and SHR rats both featured reticular structures that resembled each other. This similarity between WKY and SHR rats was also observed in TA at 12 weeks as well as AA and TA at 44 weeks. In addition, ageing induced similar variations in ELs in WKY rats as hypertension, whereas for SHR, the general arrangement and orderliness of fibres did not change significantly with ageing. In terms of the SMLs, no significant differences were observed. Sparse and regular collagen and elastin fibres with weaker signals relative to ELs were observed in both WKY and SHR rats at all three age stages.

As for the tunica adventitia, its overwhelmingly dominant fibre component, the collagen fibres, was arranged in an orderly wavy shape (figure 5). The structure was quite different from that of the internal surface (figure 4), but was generally shared by AA and TA, WKY and SHR rats, as well as the three ages. The only distinguishable difference is that the AA of 12- and 44-week-old rats have larger collagen fibre ‘waves’ than the others (figure 5).

Altogether, MPM imaging demonstrated clear differentiation and detailed depiction of the 3D microstructures of collagen and elastin fibres within the aortic wall. Compared with conventional histological section imaging, it has a spatial characterization ability, shows higher specificity, and avoids time-consuming sample preparation (involving embedding, sectioning, and staining) by directly imaging fresh tissues.

### 3.3. Hypertension-induced microtextural variations in aortic fibres quantified by 3D GLCM analysis

Despite the superiority of MPM imaging relative to conventional histological analysis, variations in aortic fibres induced by age-related hypertension cannot be fully revealed by qualitative and subjective interpretation of MPM images alone. Hence, we further performed quantitative deciphering by performing a 3D GLCM textural analysis.

#### 3.3.1. Criteria for screening and classifying 3D GLCM texture features

**Figure 6.**
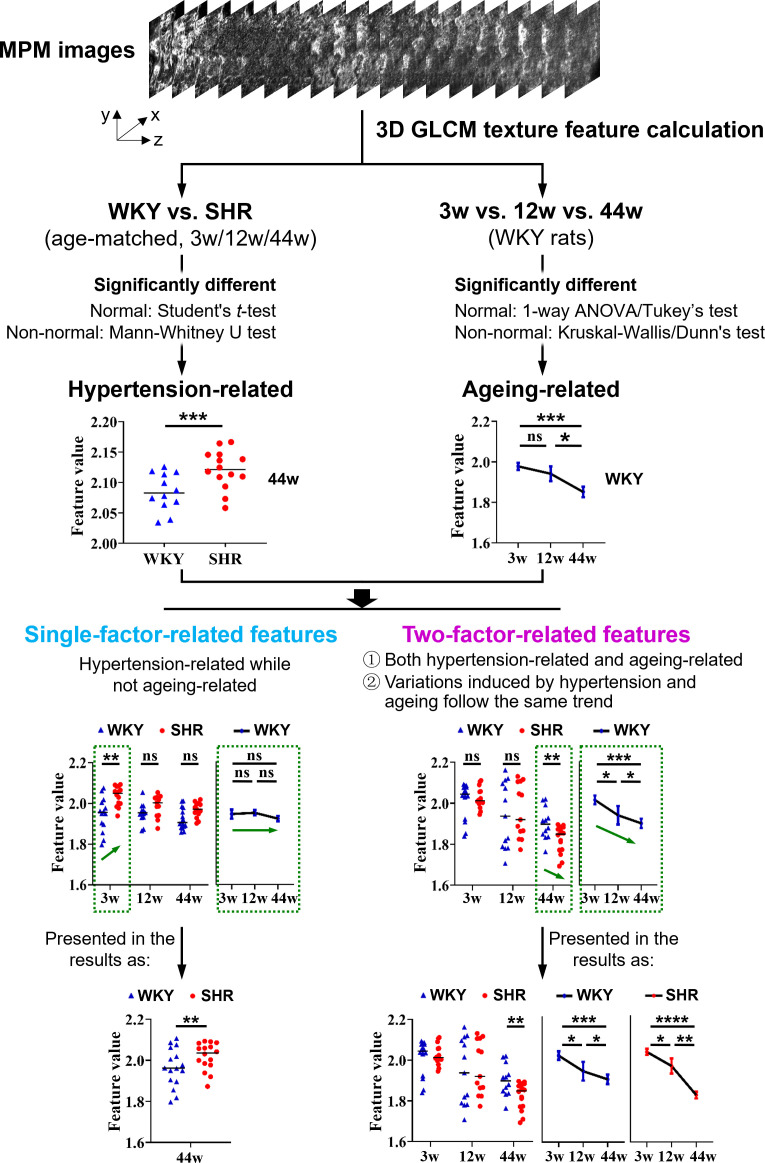
Screening and classification of three-dimensional (3D) grey-level co-occurrence matrix (GLCM) texture features. MPM, multiphoton microscopy; SHR, spontaneously hypertensive rats; WKY, Wistar–Kyoto rats; 3w, 3 weeks old; 12w, 12 weeks old; 44w, 44 weeks old. The thin black solid lines in the statistical graphics denote the medians, and the error bars represent the standard error of the mean. * (one or more): significantly different; ns: no significant difference. Green arrows indicate variation trends.

Ten 3D GLCM texture features, including *entropy*, *max probability*, *sum mean*, *energy*, *contrast*, *cluster prominence*, *cluster shade*, *correlation*, *variance* and *homogeneity*, were calculated. The definitions and descriptions of these features are provided in table 1. Based on the calculations and statistical analysis across the two strains of rats (WKY and SHR), three age stages (3-week-old, 12-week-old and 44-week-old), two aortic segments (AA and TA), two surfaces of the aortic wall (internal and external) and two types of fibres (collagen and elastin), features that hold considerable promise for revealing and quantifying hypertension-induced aortic fibre remodelling were identified. The preferential texture features generally fall into one of two categories. The first category, which we named as ‘single-factor-related features’, depicts aortal fibre remodelling merely caused by hypertension. The second category, named as ‘two-factor-related features’, reflects aortal fibre remodelling induced by both hypertension and ageing (figure 6).

Specifically, texture features that showed significant differences between SHR and WKY rats of matched age were considered hypertension-related, whereas those significantly different among WKY rats of different ages were identified as ageing-related. ‘Single-factor-related features’ simply refer to features that are hypertension-related and not ageing-related. The ‘two-factor-related features’ are defined by the following two conditions. First, the feature should be not only hypertension-related but also ageing-related. Second, the variation trend of the feature value from normal to hypertension should be the same as that from young to old, since hypertension has been found to accelerate the normal ageing process [4] (figure 6). Together, these features revealed hypertension-induced microstructural changes in the aortic fibres, and the second category further detailed hypertension-accelerated ageing. Related data and discussions on intima–media elastin fibres, intima–media collagen fibres and adventitia collagen fibres are presented in the next three subsections. Adventitial elastin fibres were not involved in the quantification because of their remarkably low content.

#### 3.3.2. Microtextural variations in intima–media elastin fibres

**Figure 7.**
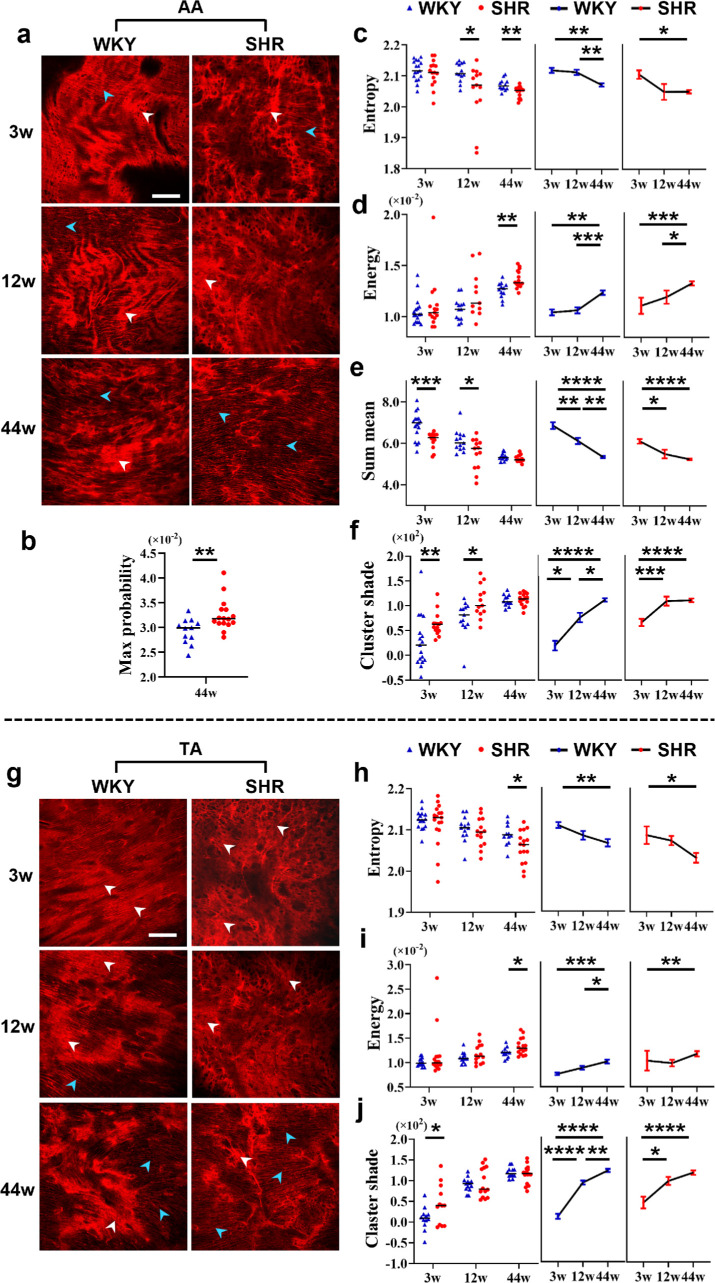
Hypertension-induced microtextural variations in intima–media elastin fibres in the abdominal aorta (AA, a–f) and thoracic aorta (TA, g–j). (a,g) Representative two-dimensional (2D) single-slice two-photon excitation fluorescence (TPEF) images obtained from spontaneously hypertensive rats (SHR) and Wistar–Kyoto (WKY) controls of different ages (3w, 3 weeks old; 12w, 12 weeks old; 44w, 44 weeks old) showing microtextural variations in (a) AA and (g) TA. White and cyan arrowheads indicate elastic laminae (ELs) and smooth muscle-rich layers (SMLs), respectively. Scale bars: 100 μm. (b–f,h–j) Three-dimensional (3D) grey-level co-occurrence matrix (GLCM) features selected for quantifying the microtextural variations: one ‘single-factor-related feature’ (b) and four ‘two-factor-related features’ (c–f) for AA, three ‘two-factor-related features’ (h–j) for TA. The thin black solid lines denote the medians. The error bars represent the standard error of the mean. *p < 0.05; **p < 0.01; ***p < 0.001; ****p < 0.0001.

For the elastin fibres within the internal surface of AA (figure 7a), there are one ‘single-factor-related feature’, *max probability* (figure 7b), and four ‘two-factor-related features’ including *entropy*, *energy*, *sum mean* and *cluster shade* (figure 7c–f). The differences between SHR and age-matched WKY controls in these features imply that hypertension-related genes or factors, or hypertension itself decreased general fibre intensity (*sum mean*, figure 7e) and increased local intensity variation (*cluster shade*, figure 7f) at prehypertension to hypertension development stages (3 and 12 weeks of age), whereas it increased the textural reproducibility (*max probability*, figure 7b) and uniformity (*energy*, figure 7d) and decreased the textural randomness (*entropy*, figure 7c) at hypertension development to stable hypertension stages (12 and 44 weeks of age). Among these features, *sum mean* and *cluster shade* mirrored EL changes described above (§3.2), whereas *energy* and *entropy* variations aligned with known effects of hypertension—EL (white arrowheads in figure 7a) fragmentation and SML (cyan arrowheads in figure 7a) fibre alignment [37,38]. Moreover, the ‘two-factor-related features’ account for up to four-fifths of all the features, providing new evidence and fine-grained details for hypertension accelerating ageing.

Hypertension- and ageing-induced changes in TA aligned with those in AA but were relatively minor. Specifically, the microstructural changes of elastin fibres within the TA internal surface were demonstrated by three ‘two-factor-related features’: *entropy*, *energy* and *cluster shade* (figure 7g–j). These features (figure 7h–j) have all been covered in the aforementioned five AA features (figure 7c–f) and show consistent variation trends between TA and AA whether across strains or ages.

Overall, a total of five texture features were identified to quantify hypertension-induced elastin fibre remodelling in aortic internal surface, and most of them are ‘two-factor-related features’. Additionally, the fibre remodelling exhibited distinct morphological patterns across age groups (demonstrated by *sum mean* and *cluster shade* at 3 and 12 weeks of age, whereas characterized by *max probability*, *energy* and *entropy* at 12 and 44 weeks of age; figure 7). These age-dependent differences probably arise from a shift in the dominant contributing factors from hypertension-related genes in the early stages to the accumulated mechanical stress caused by persistent hypertension during ageing. Altogether, these results unveiled microstructure-level details of intima–media elastin fibre reorganization induced by age-related hypertension, supporting the hypothesis that hypertension drives aortic fibre remodelling and accelerates ageing.

#### 3.3.3. Microtextural variations in intima–media collagen fibres

**Figure 8.**
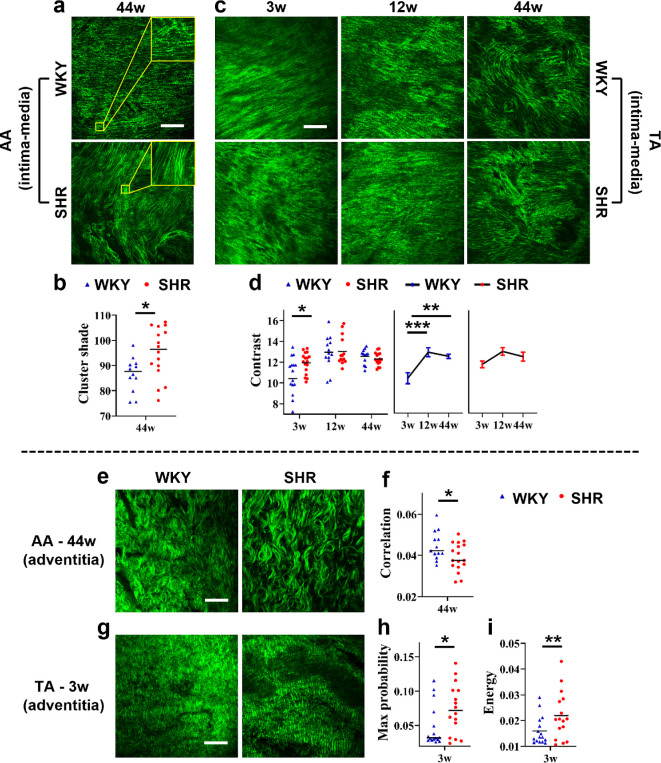
(a,b) Hypertension-induced microtextural variations in intima–media collagen fibres in the abdominal aorta (AA). (c,d) Intima–media collagen fibres in the thoracic aorta (TA). (e,f) Adventitia collagen fibres in the AA. (g–i) Adventitia collagen fibres in the TA. Note that variations in collagen fibres in AA occurred only at the stable hypertension stage (44 weeks of age [44w]), whereas variations in TA occurred only at the prehypertension stage (3 weeks of age [3w]). (a,c,e,g) Representative two-dimensional (2D) single-slice second-harmonic generation (SHG) images obtained from spontaneously hypertensive rats (SHR) and Wistar Kyoto (WKY) controls of different ages (3w, 12 weeks of age [12w] and 44w) showing microtextural variations. Scale bars: 100 μm. (b,d,f,h,i) Three-dimensional (3D) grey-level co-occurrence matrix (GLCM) texture features selected for quantifying the variations: (b) one single-factor-related feature and (d) one two-factor-related feature for intima–media collagen fibres in the AA and TA, respectively; (f) one and (h–i) two single-factor-related features for adventitia collagen fibres in the AA and TA, respectively. The thin black solid lines denote the medians. The error bars represent the standard error of the mean. *p < 0.05; **p < 0.01; ***p < 0.001.

Compared to elastin fibres within the aortic internal surface or intima–media (figure 7), hypertension-induced variations in the corresponding collagen fibres were relatively minor (figure 8a–d). There is only one ‘single-factor-related feature’, *cluster shade* (figure 8b), and one ‘two-factor-related feature’, *contrast* (figure 8d), for AA and TA, respectively. In terms of AA, collagen fibre remodelling occurred at the stable hypertension stage (44 weeks of age) and was demonstrated by an increase in *cluster shade* in SHR relative to that in WKY rats (figure 8b). This is consistent with the fact that the collagen fibres of 44-week-old SHR present increased continuity and orderliness in local texture relative to those of the WKY rats (figure 8a), as *cluster shade* reflects the local intensity variation. This variation probably stems from hypertension-induced mechanical forces that chronically stiffen the aortic wall, leading to straightening of medial collagen fibres [39,40].

For the TA, at the prehypertension stage (3 weeks of age), the collagen fibre microstructure of the tunica media had already changed (figure 8c,d). Specifically, the 3-week-old SHR exhibited reduced straightness and orderliness in fibre texture and decreased uniformity in fibre thickness, but improved fibre clarity relative to age-matched WKY controls. Similar changes were observed in 12- and 44-week-old WKY rats relative to the strain-matched 3-week-old rats (figure 8c). The variations were quantified by the increase in *contrast* (characterizing textural sharpness) and were demonstrated to accelerate ageing (figure 8d).

Collectively, hypertension-induced collagen fibre remodelling in the aortic tunica media was minor but varied between different aortic segments in terms of both morphological characteristics and age-dependent progression patterns. These differences may originate from the inherent haemodynamic heterogeneity across the aortic segments, a hypothesis that requires further validation.

#### 3.3.4. Microtextural variations in adventitia collagen fibres

Similar to AA internal surface or tunica media (figure 8a–b), the hypertension-induced collagen fibre remodelling in AA external surface or tunica adventitia was also minor and characterized by only one ‘single-factor-related feature’. In addition, the remodelling also occurred during the stable hypertension stage (44 weeks of age) (figure 8e–f). The only discrepancy is the specific texture feature. This distinction may result from inherent morphological differences between the collagen fibres of the media and adventitia. The texture feature that demonstrates AA adventitia remodelling is *correlation*, which reflects the local correlation of grey levels and decreased in 44-week-old SHR rats relative to age-matched WKY rats (figure 8f). This is consistent with the observation that collagen fibres appeared elongated in SHR with a looser and more orderly arranged texture compared to those in WKY rats (figure 8e). Mechanical stress from blood that exceeds the normal limit due to long-term hypertension may account for this variation.

Similar to the TA tunica media (figure 8c,d), remodelling of collagen fibres in the TA tunica adventitia (figure 8g–i) also occurred as early as prehypertension (3 weeks of age), which may be induced by hypertension-related genes or factors. The specific variations (figure 8g) were very similar to those of AA adventitia collagen fibres (figure 8e). However, owing to the distinct morphology of collagen fibres in tunica media and adventitia, as well as the distinct scale of collagen fibre ‘waves’ in AA adventitia and TA adventitia, totally different texture features were identified. For TA adventitia, the collagen fibre remodelling was quantified by two ‘single-factor-related features’, *max probability* and *energy*. These features characterize textural reproducibility and uniformity, respectively, and increased in 3-week-old SHR rats relative to age-matched WKY rats (figure 8h–i), which is in line with our observations (figure 8h) discussed above.

Overall, hypertension-induced collagen fibre remodelling in the aortic tunica adventitia was minor, similar to that observed in the tunica media. However, in contrast to the distinct variations in the tunica media of the AA and TA, the remodelling of collagen fibres in the AA and TA tunica adventitia seems similar. In addition, hypertension-induced remodelling of adventitia collagen fibres does not appear to significantly accelerate aortic ageing.

## 4. Discussion and conclusion

We proposed deciphering age-related hypertension from the perspective of aortic fibre remodelling by combining MPM imaging with a 3D GLCM algorithm. MPM provides aortic fibre visualization with details comparable to standard histology, while offering critical advantages that are essential for remodelling analysis. First, unlike histology, which is restricted to 2D imaging and requires destructive tissue sectioning, MPM enables direct 3D imaging leveraging its intrinsic optical sectioning ability. Second, while conventional histology relies on separate stains (Masson’s trichrome for collagen and VVG for elastin), MPM achieves simultaneous label-free visualization of collagen (via SHG) and elastin (via TPEF) with perfect spectral separation. By further integrating with 3D GLCM analysis, MPM enables quantitative colocalization studies of collagen and elastin, revealing their microtextural variations that are otherwise indiscernible from qualitative images alone. In addition to the 3D GLCM, we evaluated alternative methods for quantifying aortic fibre microstructures, such as a 3D weighted vector summation algorithm [41] for fibre orientation assessment (electronic supplementary material, figure S1a) and a pore detection algorithm for inter-fibre pore area analysis (electronic supplementary material, figure S1b). However, these methods failed to detect significant inter-group differences or showed substantial interindividual variability (electronic supplementary material, figure S1). By contrast, the 3D GLCM approach demonstrated superior performance by providing ten complementary texture features that collectively enabled the sensitive detection of subtle yet widespread hypertension-induced microstructural remodelling in the aortic wall. Therefore, we selected the 3D GLCM as the primary analytical method.

**Figure 9.**
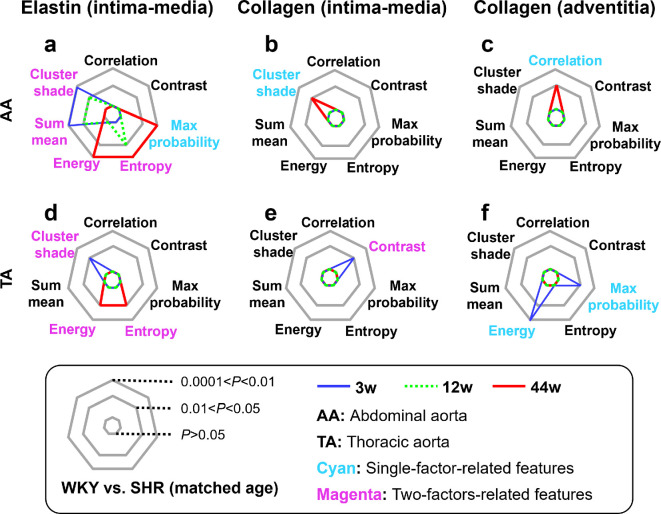
Radar plots for comparing three-dimensional (3D) grey-level co-occurrence matrix (GLCM) texture features selected for quantifying the micro-level aortic fibre remodelling in spontaneously hypertensive rats (SHR) relative to the age-matched Wistar–Kyoto (WKY) controls across three critical age stages (3w, 3 weeks old; 12w, 12 weeks old; 44w, 44 weeks old), the two aortic segments (abdominal aorta [AA] and thoracic aorta [TA]), two surfaces of the aortic wall (internal [intima–media] and external [adventitia]), and the two types of fibres (collagen and elastin). (a)–(c) Radar plots for intima–media elastin fibres, intima–media collagen fibres and adventitial collagen fibres in AA . (d)–(f) Corresponding results for TA.

Utilizing the proposed method of integrating MPM imaging with 3D GLCM analysis, microstructural remodelling of aortic fibres induced by age-related hypertension was revealed by comparing SHR with WKY controls across three critical age stages (prehypertension [3-week-old], developing hypertension [12-week-old], and stable hypertension [44-week-old]). Two aortic segments (AA and TA) and two surfaces of the aortic wall (internal [intima–media] and external [adventitia]) were systematically studied. 3D GLCM texture features characterizing aortic fibre variations merely induced by hypertension (single-factor-related features) and features quantifying variations correlated with both hypertension and ageing (two-factor-related features) were identified (summarized in figure 9).

The dominant variation induced by hypertension occurred in the intima–media elastin fibres (figure 9a,d), as they derived the highest number (five) of hypertension-related features among all three layer-specific fibre components (figure 9). Meanwhile, four of the five hypertension-related features mentioned above are also ageing-related (i.e. two-factor-related; figure 9a,d). By contrast, only one ‘two-factor-related feature’ was derived from the collagen fibres in total (figure 9b,c,e,f). Thus, the accelerating effect of hypertension on ageing may also be mainly reflected in intima–media elastin fibres. Moreover, almost consistent variations were observed in the AA and TA elastin fibres (figure 9a,d). These conclusions are consistent with established evidence that hypertension and ageing both increase aortic stiffness [4], a phenomenon primarily attributed to elastin fibre fragmentation [42]. Intima–media exhibited predominant hypertension-induced remodelling may stem from the fact that blood directly interacts with the internal surface of the aortic wall through mechanical or biochemical pathways.

Collagen fibre remodelling, although minor, is also interesting. It occurred synchronously in the intima–media and adventitia of individual aortic segments but showed significant temporal differences between segments (figure 9b,c,e,f). The aorta carries blood from the heart to the circulatory system, and fibres are the primary load-bearing components of the aortic wall [26]. Fibre remodelling in the TA generally occurs earlier relative to AA (figure 9), possibly because the TA is located closer to the heart and thus bears higher blood pressure. In addition, aortic fibre remodelling started as early as the prehypertension stage (figure 9a,d–f), which might have been triggered by genetic or other unknown factors related to hypertension and deserves further exploration. Besides, some 3D GLCM features only correlated with ageing, and some features presented opposite variation trends during hypertension versus during ageing. These features may not sufficiently elucidate age-related hypertension. Thus, we do not discuss them in this study.

Overall, by integrating MPM imaging with GLCM texture analysis, we propose a label-free, 3D and sensitive method for quantifying microstructural changes in aortic fibres. This approach revealed new details of hypertension-induced aortic remodelling, previously unrecognized aspects of how hypertension accelerates aortic ageing, and heterogeneous responses of different aortic segments to hypertension. These findings enhanced our understanding of the development of age-related hypertension and may provide new insights into its prevention and control. Moreover, the novel perspective of microstructural remodelling of aortic fibres and the proposed characterization method hold considerable promise for research on the onset and progression of various cardiovascular diseases.

However, this study had some limitations. First, the relatively small sample size may contribute to inter-sample variability. Future studies with larger cohorts are needed to further validate our findings and enhance the statistical power of the conclusions. Second, to enable en face 3D MPM imaging of the luminal surface, the aortic segments were opened longitudinally. This procedure eliminated local tensile/compressive effects, which probably differ between normotensive and hypertensive rats, as well as between young and aged rats, owing to variations in residual stress, wall thickness and lumen diameter. Nonetheless, through standardized experimental protocols and comparative analyses, we identified the distinct remodelling characteristics of aortic fibres induced by age-related hypertension. A more physiologically relevant assessment requires intravital imaging to preserve the native mechanical properties of the aorta. This technical challenge could be addressed by emerging multiphoton endoscopy techniques [43,44]. Third, inflammatory signals mediate artery stiffening [45]. Further biochemical analysis of inflammation would be valuable for unveiling the molecular mechanisms underlying aortic fibre remodelling in age-related hypertension.

## Data Availability

All data and codes used in this study are available on Mendeley [46]. Supplementary material is available online [47].
